# Effects of natural nest temperatures on sex reversal and sex ratios in an Australian alpine skink

**DOI:** 10.1038/s41598-021-99702-1

**Published:** 2021-10-11

**Authors:** Duminda S. B. Dissanayake, Clare E. Holleley, Arthur Georges

**Affiliations:** 1 Institute for Applied Ecology, University of Canberra, Canberra, ACT 2601 Australia; 2 Australian National Wildlife Collection, CSIRO, Canberra, ACT 2911 Australia

**Keywords:** Evolution, Climate sciences, Ecology

## Abstract

Altered climate regimes have the capacity to affect the physiology, development, ecology and behaviour of organisms dramatically, with consequential changes in individual fitness and so the ability of populations to persist under climatic change. More directly, extreme temperatures can directly skew the population sex ratio in some species, with substantial demographic consequences that influence the rate of population decline and recovery rates. In contrast, this is particularly true for species whose sex is determined entirely by temperature (TSD). The recent discovery of sex reversal in species with genotypic sex determination (GSD) due to extreme environmental temperatures in the wild broadens the range of species vulnerable to changing environmental temperatures through an influence on primary sex ratio. Here we document the levels of sex reversal in nests of the Australian alpine three-lined skink (*Bassiana duperreyi*), a species with sex chromosomes and sex reversal at temperatures below 20 °C and variation in rates of sex reversal with elevation. The frequency of sex reversal in nests of *B. duperreyi* ranged from 28.6% at the highest, coolest locations to zero at the lowest, warmest locations. Sex reversal in this alpine skink makes it a sensitive indicator of climate change, both in terms of changes in average temperatures and in terms of climatic variability.

## Introduction

Climate change has one of the most widespread effects on organisms across diverse ecosystems^1,2^. The big question is whether living species can adapt quickly enough to persist given current rates of climate change? Of particular concern are species whose fundamental biology is directly affected by ambient thermal regimes, particularly those whose sex is determined by temperature. Evolution is typically thought to occur slowly in comparison with ecological and demographic processes. Phenotypic plasticity not an evolutionary response, at least proximally, and is generally invoked to explain or predict species responses to rapid climate change^1,3,4^. Still, if the selection is particularly strong in the context of high genetic variability and heritability, or if Fisher's frequency-dependent selection is involved, rapid evolution under climate change is possible^5–8^.

While the process of sex determination is relatively conserved in mammals^9^ and birds^10^, reptiles exhibit many different sex-determining systems, some involving nest temperatures as the sex determining factor^11–13^. In some other species, sex is determined by an interaction between genotype (chromosomal sex) and environment^7,14–16^. In particular, sex reversal under the influence of high or low developmental temperatures has been demonstrated in two squamates. The Australian dragon lizard, *Pogona vitticeps,* has a female heterogametic ZZ/ZW system of chromosomal sex determination whereby the ZZ genotype is reversed to a female phenotype at high incubation temperatures in both the laboratory^14^ and the field^7^. The Australian skink, *Bassiana duperreyi* (our taxonomic nomenclature follows that of Hutchinson et al.^17^), has a male heterogametic XX/XY system of chromosomal sex determination whereby the XX genotype is reversed to a male phenotype at low incubation temperatures, again both in the laboratory and the field^15,18–21^. Other reptile species appear to show an underlying genetic predisposition that is over-ridden by temperature^22,23^, suggesting that the phenomenon of sex reversal could be quite widespread.

Understanding sex reversal in reptiles is essential because it has profound demographic and evolutionary consequences. Alpine *B. duperreyi* is distributed from *ca* 300 m a.s.l. to, *ca* 2020 m a.s.l in the Australian Alps of south-eastern Australia^21,24^. As an oviparous lizard, the developing embryos experience a wide range of environmental conditions, particularly high daily, seasonal, and stochastic temperature variation during their incubation period. The question arises as to what effect this has on the frequency of sex reversal and the sex ratio? What are the likely consequences of sex reversal under changing climate, and what scope does the species have to moderate climate change effects through phenotypic plasticity in the timing of nesting, nest site selection, and nest construction? To address these questions, we provide data on the relationship between fluctuating temperatures in natural nests, the frequency of sex reversal in those nests and the impact on offspring sex ratios.

## Materials and methods

### Study sites and field season

**Figure 1:**
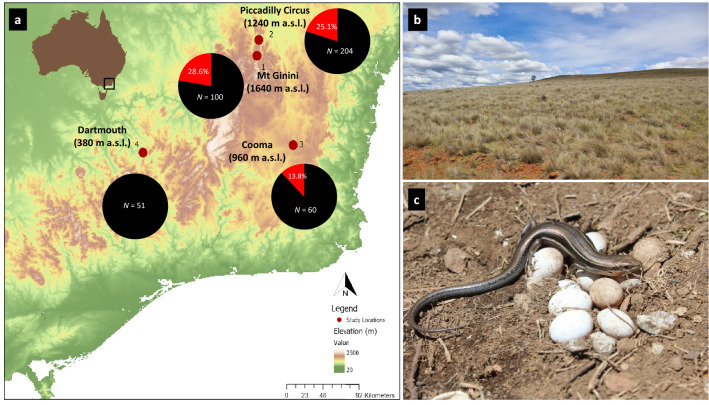
(**a**). Study locations and relative proportion of phenotypic males (black) and sex- reversed XX males (red). N = total number of eggs collected during the study period. Underlying map generated using ArcGIS 10.5.1 (http://www.esri.com) and data from the Digital Elevation Model (Geoscience Australia) made available under Creative Commons Attribution 3.0 Australia (https://creativecommons.org/licenses/by/3.0/au/legalcode, last accessed 21-Dec-20). (**b**). Typical habitat of *B. duperreyi* distribution (Cooma field location). (**c**). A typical nest of *B. duperreyi* (Location: Piccadilly Circus). All photos were taken by the first author.

Four sites along an elevational gradient were selected within the alpine region of the range of *B. duperreyi* in mainland south-eastern Australia (Fig. 1a). This series of populations is within a single substantive evolutionary lineage of the species^21^. Mount Ginini (ACT, 35° 31ʹ 29.6ʺ S 148° 46ʹ 58.7ʺ E) has the highest elevation (1640 m a.s.l.), followed by Piccadilly Circus (1240 m a.s.l., ACT, 35° 21ʹ 42.0ʺ S 148° 48ʹ 12.5ʺ E), Cooma (960 m a.s.l., NSW, 36° 26ʹ 48.6ʺ S 149° 11ʹ 40.6ʺ E) (Fig. 1b) and Dartmouth (380 m a.s.l, Victoria, 36° 31ʹ 35.9ʺ S 147° 28ʹ 53.0ʺ E) with the lowest elevation.

Due to a limited time frame and logistical constraints (significant bush fires in the field locations and adjacent areas^25^), we conducted fieldwork at Piccadilly Circus only during 2017/18 (first season). During the subsequent season of 2018/19, we completed fieldwork at all four sites. For this reason, nest temperatures with the season and associated sex reversal frequency were analysed only for Piccadilly Circus.

### Climatic data

Thermal data-loggers (iButton® model DS1921G, Maxim Integrated, San Jose, CA, USA; accuracy ± 1 °C from − 30 °C to + 70 °C; dia 17 mm, height 6.2 mm, mass 3.2 g) were placed to measure the soil temperature at the surface, 10 cm and 20 cm depth at each field location. For each location, daily projections of climatic data were obtained from a publicly available spatial data repository (SILO, Scientific Information for Landowners^26^ constructed from records provided by the Australian Bureau of Meteorology, Canberra. The data were for the period 1889 to present at a grid resolution of 0.05° latitude by 0.05° longitude (approximately 5 km × 5 km)^26^.

### Timing of breeding season and fieldwork

Female *B. duperreyi* lay clutches of eggs in communal nests (Fig. 1c). in late November to early January each year depending upon seasonal conditions. Nests are typically found under rocks in exposed areas subject to high solar radiation^27–29^. Therefore, we started our fieldwork in the first week of November to locate nests as soon as possible after laying (typically 3–7 days incubation before discovery) in both open and forested areas in both seasons. We searched thoroughly for nests from November to mid-January in each year.

### Nest characteristics and nest temperature

Each nest was marked with a plastic flag and had the GPS locations recorded. The flag was temporarily removed, and we recorded minimum nest depth (to top of the shallowest egg) and maximum nest depth (to the bottom of the deepest egg). If nest depth was less than 10 cm, one thermal data-logger (iButton® model DS1921G, Maxim Integrated, San Jose, CA, USA; accuracy ± 1 °C from − 30 °C to + 70 °C; diameter 17 mm, height 6.2 mm, mass 3.2 g) was placed in the core of the nest; if nest depth was greater than 10 cm, two iButtons were placed immediately above and below the egg mass of each nest. The iButtons were factory calibrated and were set to record the temperature at hourly intervals throughout the incubation period. We monitored nests weekly (9 weeks) for their condition, except at Dartmouth (7 weeks). We compared the data from 1997–1998 through 2006– 2007 and 2005–2006 summer seasons nest temperature and nest depth data published by Telemeco et al.^30^ using a regression model for *B. duperreyi* at the Piccadilly Circus.

### Egg collection, incubation and sample collection

After 9–10 weeks of development in the field (approximately 90% of the incubation period), we removed 415 eggs from 42 randomly selected nests and transferred them to plastic boxes in which they were buried in moist vermiculite (4 parts water to 5 parts vermiculate by weight). Each egg was separated by plastic partitions. The eggs were transported in a portable incubator set to 23 °C with high but unmeasured humidity. All experimental protocols were conducted with permission and in accordance with the procedures of Animal Ethics Committees at the University of Canberra and the CSIRO. Eggs were weighed with an electronic balance (± 0.01 g), and egg lengths and widths were measured using digital vernier callipers (± 0.01 mm). Eggs were incubated 23 °C (± 0.5 °C), which typically produces a balanced sex ratio^15^; this was a precaution only, because the eggs were harvested after the temperature is likely to exert an influence on offspring sex^15^. The boxes were gently rotated inside the incubator every couple of days, and hatchlings (hatching success was 95.2%) were removed as soon as they emerged from the egg. Sex was identified by manually everting the hemipenes of males^15,31^. Tail tips (4–5 mm) were removed with a sterile blade and the free-flowing blood drop collected onto a labelled Whatman FTA™ Elute Card (WHAWB12-0401, GE Healthcare UK Limited, UK); tail tips were collected into labelled 1.5 ml tubes containing 90% ethanol.

This the study was conducted and is reported in accordance with ARRIVE guidelines (https://arriveguidelines.org).

### Molecular detection of sex reversal

DNA was extracted from tail tips using a Gentra Puregene commercial kit (Qiagen Science, Maryland, U.S.A.) following manufacturer protocols; DNA was extracted from blood samples following manufacturer protocols. DNA purity was determined using a NanoDrop 1000 spectrophotometer (NanoDrop Technologies Inc., Wilmington, DE, USA) and quantified using the Qubit 2.0 Fluorometric Quantitation (Invitrogen, Life technologies, Sydney, N.S.W., Australia). The genotypic sex was identified using a PCR test based on seven Y-specific markers^32^. Briefly, in applying the test we used 1 × MyTaq™ HS Red mix (Bioline U.S.A. Inc. USA), 4 µM of each primer, and 25 ng of genomic DNA. The PCR cycling conditions used an initial touchdown phase to increase the specificity of amplification: denaturing at 95 °C, annealing temperature stepping down from 70 °C by 0.5 °C per cycle. This was followed by 30 cycles of 95 °C denaturing (20 s), 65 °C annealing and 72 °C extension (10 min). PCR products were visualised on a 1.5% agarose gel using SYBR Safe (Life Technologies, Carlsbad, USA). The samples that showed an amplified band for each of the seven markers are recognised as XY individuals, whereas as the samples for which a band was not amplified in all seven markers were recognised as XX individuals. The seven markers always concurred in their identification of genotypic sex, as did they in the original study published by Dissanayake et al.^32^. False negatives arising from recombination events are thus highly unlikely as they would present as some but not all markers detecting the presence of a Y chromosome. No XY females were observed, another indication that recombination and/or mutation involving these loci is negligible and has not affected the accuracy of genotypic sex assignment. Phenotypic male lizards showing genotype–phenotype discordance were classified as sex-reversed^21,32^. All molecular sex tests were conducted blind to the phenotypic sex of the individuals.

### Analysis

**Figure 2:**
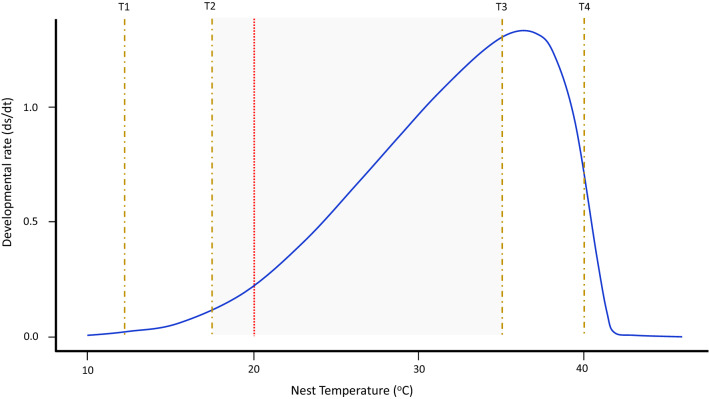
The Dallwitz-Higgins model^35^ applied to *Bassiana duperreyi* nest data. T1 and T4 are the lower and upper absolute lethal limits, outside which even brief exposure causes embryo death. T2 and T3 are the constant-temperature lethal limits, outside which a temperature held constant throughout incubation will cause embryo death or gross abnormality. Temperatures in the sublethal ranges T1 –T2 and T3 –T4 will support embryonic development (Optimal Thermal Range indicating the shaded area), provided exposure is for a part of each day only, but the duration of vulnerability that can be tolerated will decline as one move to extremes. Red broken line denotes the sex reversal threshold for *B. duperreyi* according to Shine et al.^15^.

Results are presented as means ± standard errors unless otherwise indicated. Correlation and regression analysis were used to describe relationships between variables, and the Student's t-test used to compare mean weekly air and nest temperatures. To analyse the relationship between natural thermal regimes and sex ratio of *B. duperreyi*, we corrected nest temperatures to constant temperature equivalents (CTE)^33,34^ using the reaction norm for development rate against temperature estimated using the developmental model developed by Dallwitz and Higgins^35^ (Fig. 2; Figure S1A). Sexual fate is sensitive to temperature in a wide range of reptiles during the middle third of incubation^33,34^. This has not been established for *Bassiana duperreyi*, but we nevertheless restricted our modelling to the middle third of development. We estimated the middle third of development by summing development as a function of temperature in small but finite increments, back from the point of hatching. The likelihood of reversal was based on two criteria: raw temperature and temperature corrected for developmental rate (CTE). Data analyses were performed in R (R Core Team, 2017) and GraphPad Prism version 9 for Windows (GraphPad Software, La Jolla California USA).

Simple linear regression was used to determine the relationship between the frequency of sex-reversed hatchlings in each population with respect to elevation and to characterise the trends in mean nests temperature, yearly mean maximum (Tmax), and yearly mean minimum temperature (Tmin) against elevation in each field location. Pearson correlation was used to evaluate the magnitude and direction of the association between the frequency of sex reversal and climatic variables [Tmax, Tmin, total rain (mm), evaporation (mm), radiation (Mj/m^2^) and vapor pressure (hPa)] including elevation recorded at each field site. We used a t-test to compare the sex reversal frequency of adult males (Dissanayake et al.^21^) with the sex reversal frequency our nests.

## Results

### Climate data

**Figure 3:**
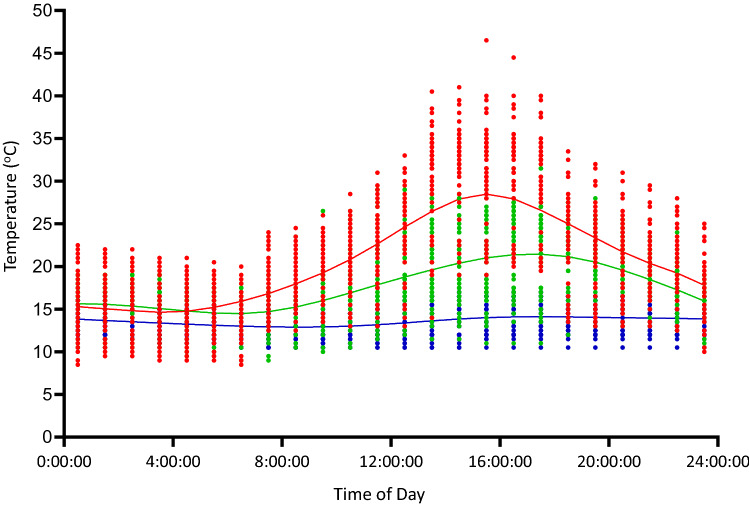
The diurnal variation of hourly soil temperature at the Picadilly circus field station. LOWESS curve fitted (solid lines) with a smooth curve to aid visual interpretation, (red): soil surface, green: at the depth of 10 cm and blue: at depth of 20 cm (blue). The minimum amplitude of the soil temperature variation approached in 20 cm depth. The daily variation of soil temperature showed a sinusoidal pattern, and the soil temperature decreased with the increase of the soil depth at all field stations. The order of the measured soil temperatures from high to low is: T0 cm > T10 cm > T20 cm.

**Figure 4:**
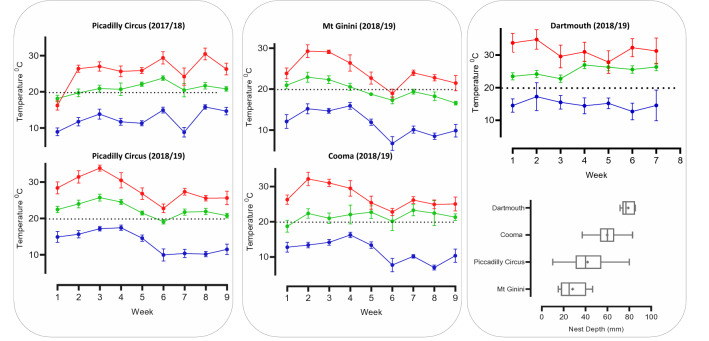
Weekly mean temperatures in the core of *Bassiana duperreyi* nests in each field locations (green) and the mean nest depth in each field location (right bottom). Red denotes Tmax and blue denotes Tmin.

Thermal profiles showed considerable diel variation at each field location (Fig. 3). Soil temperatures on the ground surface fluctuated the most, reaching maximum temperature (Mt Ginini, 45.5 °C; Piccadilly Circus first season, 46.5 °C; Piccadilly Circus second season, 46.0 °C; Cooma, 42.1 °C; Dartmouth, 47.5 °C) during the day (1330–1730 h) and dropping to low level (Mt Ginini, 4.0 °C; Piccadilly Circus first season, 8.5 °C; Piccadilly Circus second season, 6.5 °C; Cooma, 7 °C; Dartmouth 12.5 °C) at night (2330–0400 h) (ANOVA, F_4, 6521_ = 279.4, *P* < 0.0001). The soil temperature at 20 cm, the deepest we monitored, showed the least fluctuation at all locations (see Fig. 3). Nests intermediate in depth between these two extremes (surface to 20 cm) showed intermediate diel fluctuations. At all the field locations, lower mean and lower minimum temperatures and less diel thermal variation occurred at 20 cm depth than the soil surface. Monthly mean air temperatures were averaged over the skink active months (i.e. early November to late February, a 16 week period) in each year to reveal a warming trend between 1889 to 2019 (Figure S2-Mt Ginini: F_1,517_ = 523.9, *P* < 0.0001, R^2^ = 0.50; Piccadilly Circus: F_1,517_ = 539.6, *P* < 0.0001, R^2^ = 0.51; Cooma: F_1,517_ = 537.9, *P* < 0.0001, R^2^ = 0.50; Dartmouth: F_1,517_ = 627.5, *P* < 0.0001, R^2^ = 0.54). Air temperatures were consistently cooler at higher elevational locations than the lower elevations, which inversely correlated with elevation when *B. duperreyi* eggs were incubation (F_1,22_ = 4.795, *P* < 0.039, R^2^ = 0.17). The weekly mean Tmax and Tmin showed that temperatures fluctuated substantially during the 9-week eggs incubation period (Fig. 4). The highest mean rainfall events (17.0 ± 23.87 mm) were recorded at the Piccadilly Circus in the first week of egg incubation period in the first season. The highest mean rainfall events (5 ± 11.85 mm) were recorded at Mt Ginini during the third week of *B. duperreyi* egg incubation. The highest rainfalls were recorded during the fourth week of the egg incubation period at Cooma (5 ± 6.3 mm) and Dartmouth (1 ± 0.99 mm). Rainfall among the four sample locations were statistically different (Kruskal–Wallis; H = 16.83, *P* < 0.05).

### Timing of breeding season and nest search protocols

In Piccadilly Circus during first season, female *B. duperreyi* laid their first eggs in the first week of December and 39 nests were found (only 35 monitored). In the second season (2018/19) females laid their eggs in early January and 26 nests were found (only 14 monitored). In the second season, in early January we found 11 nests at Mt Ginini (only nine were monitored) and nine nests at Cooma (only eight monitored). In the second week of December four nests were found (all monitored) at Dartmouth. Overall, we observed 1335 eggs in 89 nests at the four locations (Figure S3). A total of 84 nests survived the natural incubation period; five nests (3 from Piccadilly Circus and 2 from Mt Ginini) loss their eggs for unknown reasons.

### Nest characteristics and nest temperature

**Figure 5:**
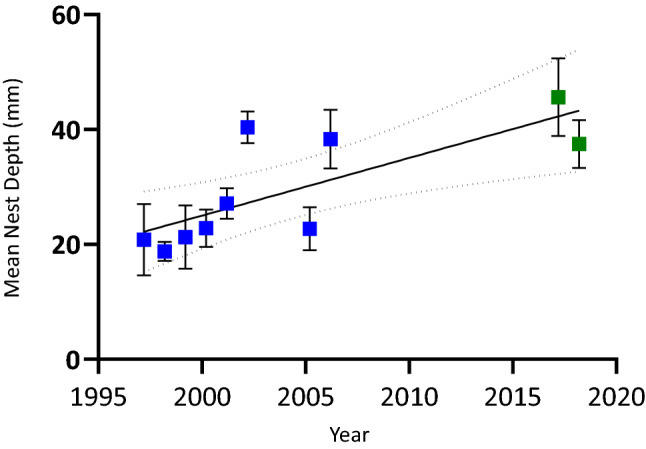
Long-term variation of mean nest depth of *Bassiana duperreyi*. The data from 1997–1998 through 2006– 2007 and 2005–2006 seasons nest depth data (blue) (Telemeco et al.^30^) and current study (green) (2017/18 and 2018/2019).

Nests were typically constructed beneath rocks (98%), though some were found associated with logs (2%). All nests were deposited in open grassland and received direct sunlight at the surface for a large proportion of each day. Once the rock was removed, nests were typically partially buried in the soil (a few eggs were visible without disturbance) (91%) with a vertical nest chamber; some nests were completely buried (eggs well covered by soil) (7%) and few were found on top of the soil (not buried) (2%). Lengths of the rocks used for nesting averaged 28.8 ± 9.5 cm for Mt Ginini, 32.8 ± 20.7 cm for Piccadilly Circus, 38.6 ± 16.8 cm for Cooma, and 32.00 ± 9 cm for Dartmouth. Rock length differed significantly with location (F_3,87_ = 5.71, *P* < 0.0001), decreasing with elevation (F_1,87_ = 11.94, *P* < 0.001, R^2^ = 0.12). Nests were shallower in the first season compared to the second season in Piccadilly Circus (t = 3.7, df = 61, *P* < 0.05). At Piccadilly Circus, females constructed progressively deeper nests over past 22 years when combining our data (2017 to 2019) an those of Telemeco et al.^30^ (1997 to 2007) (F_1,8_ = 11.36, *P* < 0.05, R^2^ = 0.58; Fig. 5). Of the 89 nests we located, 69 (71.91%) nests were communal i.e., contained more than nine eggs which according to Radder and Shine^36^ is the maximum clutch size, Radder and Shine 2007). Nest depth varied from 1 to 85 mm. The deepest nests were recorded at the lowest elevational location of Dartmouth (79 ± 6.1 mm). The shallowest nests (i.e., 15 mm) were found at the highest elevational of Mt Ginini (28.8 ± 11.7 mm) (Fig. 4). Nest depth was inversely related to elevation (F_1,86_ = 39.80, *P* < 0.0001, R^2^ = 0.32; Figure S4).

The lowest (19.67 ± 0.63 °C) and the highest (25.59 ± 1.25 °C) mean nest temperatures were recorded at Mt Ginini and Dartmouth, respectively. During nest incubation at Piccadilly Circus, mean nest temperature was lower (21.6 ± 1.41 °C) in the first season than in the second (22.2 ± 0.22 °C) (F_8, 410_ = 88.59, df = 8, *P* < 0.0001). The highest nest temperature (47.5 °C) and the highest mean daily range temperature (19.1 ± 2.30 °C) were recorded at the highest elevational location, Mt Ginini.

Most nests experienced high mean temperatures and a considerable diel range of temperatures in all field locations. Mean daily temperatures experienced by the eggs differed among nests in highest elevation, Mt Ginini (18.76–20.57 °C) to lowest elevation Dartmouth (23.89–27.39 °C), as did mean maxima in Mt Ginini (35.8–27.8 °C) and minima (10.6–13.5 °C), and mean maxima in Dartmouth (34.67–38.67 °C) and minima (15.25–17.75 °C). The nests showed significant differences in mean nest temperature maxima in all locations except Dartmouth, but mean nest temperature minima shows a significant difference in all locations (Table 1).

**Table 1:** Descriptors of nest temperatures of *B. duperreyi* in four locations. Nest temperature data were continuous temperatures taken by data loggers at one-hour intervals throughout incubation (9 weeks in Mt Ginini, Piccadilly Circus, Cooma) (7 weeks in Dartmouth).

Location	Mean nest temperature	Mean daily nest temperature range °C	Mean nest temperature maxima		Mean nest temperature minima	
			Temperature range °C	ANOVA	Temperature range °C	ANOVA
Mt Ginini	19.67 ± 0.63 °C	18.76–20.57	27.8–35.8	F_8,549_ = 15.18, *P* < 0.0001	10.6–13.5	F_8,558_ = 7.69, *P* < 0.0001
Piccadilly Circus I season	21.61 ± 1.41 °C	16.98–23.56	22.38–33.18	F_34,1834_ = 19.71, *P* < 0.0001	12.25–17.99	F_34,1832_ = 14.25, *P* < 0.0001
Piccadilly Circus II season	22.20 ± 0.22 °C	19.18–23.76	26.71–35.09	F_13,770_ = 19.30, *P* < 0.0001	15.61–17.72	F_13,756_ = 2.89, *P* < 0.0001
Cooma	22.1 ± 1.56	19.58–23.59	25.1–32.9	F_7,432_ = 13.02, *P* < 0.0001	14.5–18	F_7,440_ = 14.27, *P* < 0.0001
Dartmouth	25.59 ± 1.25 °C	23.89–27.39	34.67–38.67	F_4, 225_ = 2.63, *P* = 0.035	15.25–17.75	F_4,230_ = 9.52, *P* < 0.0001

Mean weekly nest temperature was correlated with mean weekly air Tmax (R^2^ = 0.42–0.74, *P* < 0.05) and Tmin (R^2^ = 0.70–0.93, *P* < 0001) at all field locations. The significant warming trend was recorded during the incubation weeks in first season at the Piccadilly Circus (F_1,300_ = 31.74, *P* < 0.001, R^2^ = 0.09), but a significant cooling trend was recorded in the second season (F_1,124_ = 52.63, *P* < 0.001, R^2^ = 0.29). In the second season a significant cooling trend was also recorded at Mt Ginini (F_1,79_ = 132.3, *P* < 0.001, R^2^ = 0.62). Whereas at Cooma and Dartmouth showed no significant trend as the season progressed.

**Figure 6:**
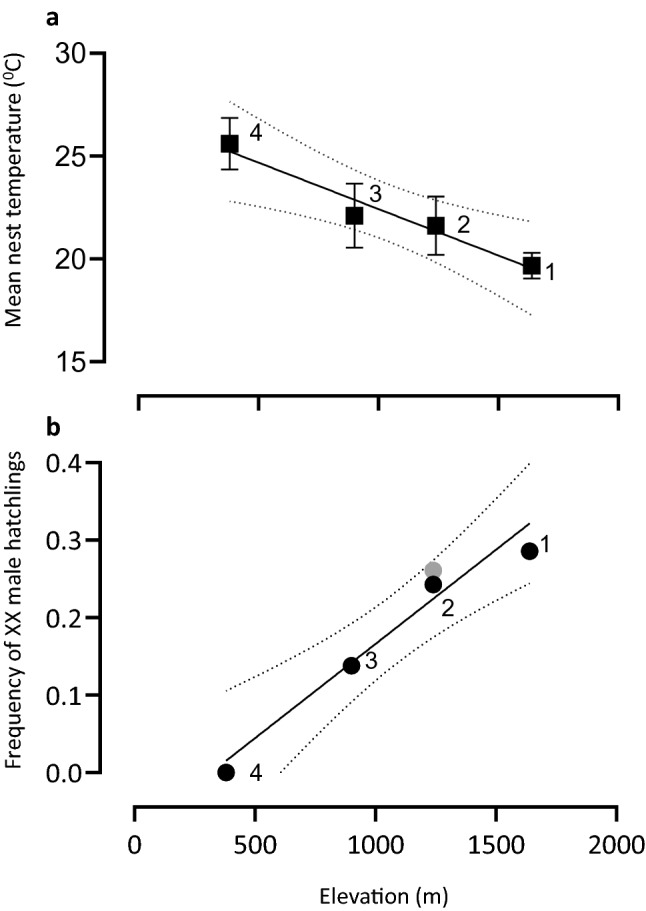
The frequency of sex reversal in nests *Bassiana duperreyi*. (**a**) Linear regression of mean nest temperature in each field location (F_1,2_ = 41.71, *P* = 0.023; R^2^ = 0.95). (**b**). The trend in sex reversal frequency of *B. duperreyi* with the elevation (F_1,3_ = 41.71, *P* < 0.05; R^2^ = 0.95). The number indicates field locations, as indicated in Fig. 1. Grey circles indicate first season data (2017/18) and black circles indicate second season data (2018/19). Broken lines denote the 95% confidence interval, and significance was assumed if *P* < 0.05.

**Figure 7:**
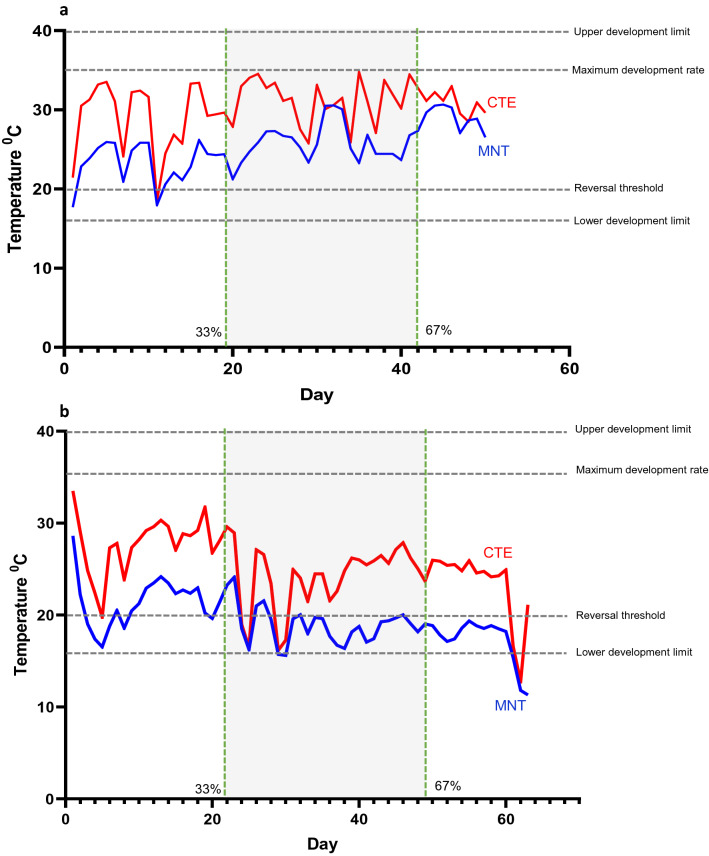
Temperature trace for the core of a nest of *Bassiana duperreyi* showing traces for the mean and the constant-temperature equivalent (CTE) for the Dallwitz-Higgins model^34,35^. (**a**). The nest produced only XYmale and XXfemale offspring, nest location: Dartmouth. (**b**). The nest produced XYmale, XXmale and XXfemale offspring, nest location: Mt Ginini. Shaded area: expecting to sex reversal happening during the incubation period. The threshold for sex determination (20 °C; Shine et al.^15^) and the thermosensitive period lies between lower and upper limit of development. Note that the thermosensitive period does not correspond to the middle third of incubation, either in position or duration, owing to the nonstationary trend in temperatures with season.

Mean nest temperatures during the incubation period were inversely correlated with elevation (F_1,2_ = 41.71, *P* < 0.05, R^2^ = 0.95) (Fig. 6a). The highest and lowest mean daily CTE were recorded at Dartmouth (30.35 ± 0.12 °C) and Mt Ginini (26.2 ± 1.98 °C), respectively. The mean daily CTE was significantly inversely correlated with elevation (F_1,74_ = 11.39, *P* < 0.001, R^2^ = 0.17). When the CTE dropped below the 20 °C (the threshold for reversal, Shine et al.^15^) during the thermosensitive period, for even for a short time during the incubation period, sex reversal was observed (Fig. 7 and Figure S1 B-F).

Nest depth was not significantly associated with mean nest temperature (F_1,84_ = 0.081, *P* = 0.77) at any location. Examination of mean nest temperatures suggested a slight warming trend at Piccadilly Circus during last 22 years, but this was not statistically significant (F_1,8_ = 3.49, *P* = 0.09). Nests that produced sex-reversed hatchlings had mean daily nest temperatures cooler than nests that did not produce sex reversed hatchlings. However, mean weekly temperature regimes for these two categories of nests were not significantly different at any field location (Table S1).

### Genotypic sex identification and the frequency of sex reversal

During the study period, we collected a total of 415 eggs from the natural nests. The hatching success rate was 95.2%. Therefore, a total of 395 hatchlings were able to be phenotypically identified. Of them, 262 (66.3%) were phenotypic males and 133 (33.6%) were phenotypic females. The sex ratio (Phenotypic male: female) of each location is as follows; Mt Ginini (2018/19) = 2.3 : 1; Picadilly Circus (2017/18 and 2018/19) = 2.27 : 1; Cooma (2018/19) = 1.38 : 1 and Dartmouth (2018/19) = 1.14 : 1, yielding a male-biased sex ratio in the high elevation sites, where adult sex reversal has been previously identified by Dissanayake et al.^21^ (chi-squared test with Yates correction, χ2 = 4.05, df = 1, *P* < 0.05). A total of 59 (7.03%) phenotypically male hatchlings were sex-reversed. The highest frequency of sex reversal in hatchlings was recorded at the highest elevation site (28.6%, Mt Ginini, 1,640 m a.s.l.), and zero sex reversal was observed at the lowest elevation (Dartmouth, 380 m a.s.l) (Fig. 1a). This observation concorded with the previous study has been contacted for adult individuals (see Dissanayake et al.^21^). The frequency of sex reversal was positively correlated with elevation (F_1,3_ = 41.71, *P* < 0.05; R^2^ = 0.95) (Fig. 6b) and each population showed a negative correlation with mean nest temperature (Pearson’s correlation coefficient r = − 0.95, *P* < 0.05); Tmax (R2 = 0.99, *P* < 0.05) was significantly negatively correlated with sex reversal frequency (Table S2). When we compared the current study with Dissanayake et al.^21^ for adult sex reversal frequency, the frequency of sex reversal in the nest was higher than the sex-reversed adult in the same field locations, but not significantly (*P* = 0.36). However, both hatchlings and adults (Dissanayake et al.^21^) rates of sex reversal frequency positively correlate with their respective elevation (F_1,2_ = 15.17, *P* < 0.005; R^2^ = 0.77) (Figure S5) (see also Dissanayake et al.^21^).

## Discussion

Over the last two decades, many field studies and laboratory experiments have been conducted to understand *Bassiana duperreyi* nesting ecology and phenotypic plasticity^29,30,37–40^. However, the consequences of cold nest temperatures on sex reversal in *B. duperreyi* have been little studied in the wild (but see Holleley et al.^18^). We show that sex reversal in natural nests of this species is common at elevations above 900 m a.s.l. The frequency of sex reversal in nests ranges from 28.6% at the highest, coolest location (Mt Ginini, 1,640 m a.s.l., mean nest temperature 19.7 ± 0.62 °C) to zero percent at the lowest, warmest mean nest temperature recorded location (Dartmouth, 380 m a.s.l., 25.6 ± 1.25 °C). Thus, both hatchlings (this study) and adults as observed by Dissanayake et al.^21^ exhibit a comparable relationship between the frequency of sex reversal and elevational variation in ambient temperatures*.* These substantial levels of sex reversal in nests and the adult population, derived from the rates of sex reversal we have observed in the nest, indicate that the sex-reversed phenotype is an important component of the demography of this species.

How sex reversal will come to influence demographic processes that govern the persistence of local populations in the Australian high country requires additional information on the fertility of sex-reversed individuals. The presence of sex-reversed adults^21^ at a similar rate that observed in the nest (this study) establishes the viability of sex-reversed individuals. But are they fertile? If the *B. duperreyi* sex reversal yields infertile male individuals, then males of the species are subject to latent mortality—a significant component of the population could comprise viable but infertile males. As these are generated by reversal of XX individuals otherwise destined to be females, the effective population size, which depends on reproductive female number, is drawn down numerically. It is also drawn down because many of the remaining XX females will potentially mate with the infertile males to no effect, and this proportion of females will not contribute to effective population size. Together, this could lead to a rapid population decline in effective population size and local extinction should there be a sequence of episodic cold seasons or cold spells at a time when the embryos in the nests are thermosensitive. In contrast, if the sex-reversed males are both viable and fertile, then sex reversal under cooler temperatures will lead to an overproduction of males and, in the context of other evolutionary and phenotypic responses, Fisher's frequency-dependent selection^41,42^ may be invoked in support of traits that bring the population sex ratio back to equilibrium. This will manifest as a reduction in the sensitivity of sex to temperature or reduction of the frequency of the Y chromosome in the population or both and, potentially, its loss^7,21,43,44^. Suppose for example, a future change in climate or annual weather is sudden, such that the species is subject to substantial sex reversal but unable to evolutionarily adjust the male-biased sex ratio. In that case, effective population size will decline, again putting local populations at greater risk of extinction. Clearly, under either scenario, sex reversal has profound implications for the demography of *B. duperreyi* at the high elevation distributional limits of its range whether or not sex-reversed males are fertile.

The above impacts of sex reversal can be ameliorated if the frequency of sex reversal can be constrained by phenotypic responses. In particular, nest site choice and nesting phenology are potentially both important in ameliorating the frequency of sex reversal. We recorded that *B. duperreyi* selected open grassland and 98% of rock substrate at all field locations for laying their eggs, similar to the findings of previous studies^29,30^. We show that the consequence of egg laying in exposed sites is a high diel variance in nest temperature. *B. duperreyi* typically select nesting sites under rocks compared to other alpine skinks that live at the same field location that typically nest in logs and rotting organic material^29^. Furthermore, *B. duperreyi* appear to select nest sites on both thermal averages and high thermal variability both of which have measurable effects on hatchling phenotype^40^. Adjusting for diel fluctuations, we found that when the CTE dropped below the 20 °C (the threshold for reversal, Shine et al.^15^) during the thermosensitive period, for even for a short time during the incubation period, sex reversal occurred (Fig. 7). Therefore despite the observed temperature fluctuations in nests, extreme cold temperature events could lead to 100% sex reversal of the XX genotype in the alpine populations at the highest elevations.

When considering the alpine populations, *B. duperreyi* embryo development occurred under high thermal fluctuations and some nests reached beyond the embryo physiological limits for brief periods. The high level of diel fluctuation of nest temperature leads some nests reaching both upper and lower limits in development temperatures. At Mt Ginini (i.e., highest elevational location for this study), four nests show that their temperature exceeded both the maximum and minimum temperature for embryo survival during the incubation period (see Table 1). However, we observed that these nests survived and successfully completed their natural embryonic development to hatchling. Therefore, a short period of exposure to extreme temperature events did not affect egg survivorship but may well have influenced the frequency of sex reversal.

The embryonic survivorship under the current increasing global temperature trend is a significant challenge for oviparous species globally. The current trend in global warming inescapably will increase the nest temperatures of many oviparous reptiles ^30,46,47^. Therefore, natural selection should favour potential nesting sites, nest phenology and other maternal behaviors that enhance embryonic survivorship. Egg laying of *B. duperreyi* shifted temporally, with the nesting season starting from mid-November to early December, confirming a similar observation by Telemeco et al.^30^. However, in our second season (2018/19), females initiated egg laying in early January, as they did in 1968^27^. *B. duperreyi* appear to shift their egg laying from year to year, presumably in response to variation in natural conditions conducive to successful incubation and emergence (see also Pengilley^27^). Incubation conditions, and so timing or egg laying, also have strong effects on offspring phenotypes^15,48^ and sex determination, including sex-reversal. In addition to altering the timing of nesting, *B. duperreyi* has progressively been digging deeper nests over the last two decades (Fig. 5 and see Telemeco et al.^30^), likely to reduce exposure to extreme daily temperatures and have a complex impact on average nest temperatures depending upon soil composition and structure.

We have shown a significant relationship between the frequency of sex reversal in hatchlings and nest temperatures during their natural incubation period. Our work establishes and quantifies current elevational trends in the frequency of sex reversal in response to cold temperatures both in the nests and in the adult population^21^. This forms a baseline for examining the effects of climate change, and in particular climate warming. On the basis of the trends in primary (egg) and operational (adult) sex ratio with elevation, presumably driven by associated variation in thermal conditions, we expect to see populations without sex reversal, that is, governed by GSD, to increase in elevation as the climate warms. At the highest altitudes, if climate change is accompanied by increased variability, this will potentially disrupt the demographic processes on a local scale, leading to local population crashes and local extinction events, driven by fluctuating frequency of sex reversal. Temperature induced sex reversal in *B. duperreyi* and its demographic consequences likely make this species a very sensitive indicator of climate change. Monitoring changes in the frequency of sex reversal through time along our elevational gradient and at the species highest distributional limits is recommended as one option for assessing the impacts of climate change on the biota of the Australian high country.

## Supplementary Information

Supplementary Information.

## Data Availability

All data and materials are presented in the main paper and supplementary information. The Scientific Information for Land-Owners' data used in this paper can be accessed at https://www.longpaddock.qld.gov.au/silo/. The datasets generated and analysed during the current study are available from the corresponding author on reasonable request.

## References

[1] Parmesan C (2006). Ecological and evolutionary responses to recent climate change. Annu. Rev. Ecol. Evol. Syst..

[2] Peñuelas J (2013). Evidence of current impact of climate change on life: a walk from genes to the biosphere. Glob. Change Biol..

[3] Chevin L-M, Lande R, Mace GM (2010). Adaptation, plasticity, and extinction in a changing environment: towards a predictive theory. PLOS Biol..

[4] Oostra V, Saastamoinen M, Zwaan BJ, Wheat CW (2018). Strong phenotypic plasticity limits potential for evolutionary responses to climate change. Nat. Commun..

[5] Conover DO, Voorhees DAV (1990). Evolution of a balanced sex ratio by frequency-dependent selection in a fish. Science.

[6] Hoffmann AA, Sgrò CM (2011). Climate change and evolutionary adaptation. Nature.

[7] Holleley CE (2015). Sex reversal triggers the rapid transition from genetic to temperature-dependent sex. Nature.

[8] Kopp M, Matuszewski S (2014). Rapid evolution of quantitative traits: theoretical perspectives. Evol. Appl..

[9] Graves JAM (1995). The evolution of mammalian sex chromosomes and the origin of sex determining genes. Philos. Trans. R. Soc. Lond. B. Biol. Sci..

[10] Smith CA, Roeszler KN, Hudson QJ, Sinclair AH (2007). Avian sex determination: what, when and where?. Cytogenet. Genome Res..

[11] Bull JJ (1980). Sex determination in reptiles. Q. Rev. Biol..

[12] Deeming DC (1988). Environmental regulation of sex determination in reptiles. Philos. Trans. R. Soc. Lond. B Biol. Sci..

[13] Janzen FJ, Paukstis GL (1991). Environmental sex determination in reptiles: ecology, evolution, and experimental design. Q. Rev. Biol..

[14] Quinn AE (2007). Temperature sex reversal implies sex gene dosage in a reptile. Science.

[15] Shine R, Elphick MJ, Donnellan S (2002). Co-occurrence of multiple, supposedly incompatible modes of sex determination in a lizard population. Ecol. Lett..

[16] Wiggins JM, Santoyo-Brito E, Scales JB, Fox SF (2020). Gene dose indicates presence of sex chromosomes in collared lizards (*Crotaphytus collaris*), a species with temperature-influenced sex determination. Herpetologica.

[17] Hutchinson MN (1990). Immunological relationships and generic revision of the australian lizards assigned to the genus *Leiolopisma* (Scincidae, Lygosominae). Aust. J. Zool..

[18] Holleley CE, Sarre SD, O’Meally D, Georges A (2016). Sex reversal in reptiles: reproductive oddity or powerful driver of evolutionary change?. Sex. Dev..

[19] Quinn AE (2009). Isolation and development of a molecular sex marker for *Bassiana duperreyi*, a lizard with XX/XY sex chromosomes and temperature-induced sex reversal. Mol. Genet. Genom..

[20] Radder RS, Quinn AE, Georges A, Sarre SD, Shine R (2008). Genetic evidence for co-occurrence of chromosomal and thermal sex-determining systems in a lizard. Biol. Lett..

[21] Dissanayake DSB, Holleley CE, Deakin JE, Georges A (2021). High elevation increases the risk of Y chromosome loss in alpine skink populations with sex reversal. Heredity.

[22] Capel B (2017). Vertebrate sex determination: evolutionary plasticity of a fundamental switch. Nat. Rev. Genet..

[23] Whiteley SL, Georges A, Weisbecker V, Schwanz LE, Holleley CE (2021). Ovotestes suggest cryptic genetic influence in a reptile model for temperature-dependent sex determination. Proc. R. Soc. B Biol. Sci..

[24] Cogger, H. *Reptiles & Amphibians*. (CSIRO Publishing, 2018).

[25] Godfree RC (2021). Implications of the 2019–2020 megafires for the biogeography and conservation of Australian vegetation. Nat. Commun..

[26] Jeffrey SJ, Carter JO, Moodie KB, Beswick AR (2001). Using spatial interpolation to construct a comprehensive archive of Australian climate data. Environ. Model. Softw..

[27] Pengilley, R. Systematic relationships and ecology of some lygosomine lizards from southeastern Australia. (PhD Thesis, Australian National University, 1972).

[28] Shine R (1995). A New Hypothesis for the evolution of viviparity in reptiles. Am. Nat..

[29] Shine R, Harlow PS (1996). Maternal manipulation of offspring phenotypes via nest-site selection in an oviparous lizard. Ecology.

[30] Telemeco RS, Elphick MJ, Shine R (2009). Nesting lizards (*Bassiana duperreyi*) compensate partly, but not completely, for climate change. Ecology.

[31] Harlow PS (1996). A harmless technique for sexing hatchling lizards. Herpetol. Rev..

[32] Dissanayake DSB (2020). Identification of Y chromosome markers in the eastern three-lined skink (*Bassiana duperreyi*) using in silico whole genome subtraction. BMC Genom..

[33] Georges A (1989). Female turtles from hot nests: is it duration of incubation or proportion of development at high temperatures that matters?. Oecologia.

[34] Georges A, Limpus C, Stoutjesdijk R (1994). Hatchling sex in the marine turtle *Caretta caretta* is determined by proportion of development at a temperature, not daily duration of exposure. J. Exp. Zool..

[35] Dallwitz, M. J. & Higgins, J. P. *User’s Guide to DEVAR. A computer program for estimating development rate as a function of temperature*. (Canberra, Division of Entomology, CSIRO, 1978). 10.25919/rsxt-5e19.

[36] Radder RS, Shine R (2007). Why do female lizards lay their eggs in communal nests?. J. Anim. Ecol..

[37] Du W-G, Shine R (2010). Why do the eggs of lizards (*Bassiana duperreyi*: Scincidae) hatch sooner if incubated at fluctuating rather than constant temperatures?. Biol. J. Linn. Soc..

[38] Elphick MJ, Shine R (1998). Longterm effects of incubation temperatures on the morphology and locomotor performance of hatchling lizards (*Bassiana duperreyi*, Scincidae). Biol. J. Linn. Soc..

[39] Shine R (1999). Egg-laying reptiles in cold climates: determinants and consequences of nest temperatures in montane lizards. J. Evol. Biol..

[40] Shine R, Elphick MJ, Harlow PS (1997). The influence of natural incubation environments on the phenotypic traits of hatchling lizards. Ecology.

[41] Düsing, K. *Die regulierung des geschlechtsverhältnisses bei der vermehrung der menschen, thiere und pflanzen*. (Fischer, 1884).

[42] Fisher, R. A. *The Genetical Theory of Natural Selection*. xiv, 272 (Clarendon Press, 1930). 10.5962/bhl.title.27468.

[43] Bull JJ (1981). Evolution of environmental sex determination from genotypic sex determination. Heredity.

[44] Schwanz LE, Georges A, Holleley CE, Sarre SD (2020). Climate change, sex reversal and lability of sex-determining systems. J. Evol. Biol..

[45] Georges A (1992). Thermal-characteristics and sex determination in field nests of the pig-nosed turtle, *Carettochelys insculpta* (Chelonia, Carettochelydidae), from northern Australia. Aust. J. Zool..

[46] Du WG, Shine R, Ma L, Sun BJ (2019). Adaptive responses of the embryos of birds and reptiles to spatial and temporal variations in nest temperatures. Proc. R. Soc. B Biol. Sci..

[47] Janzen FJ (1994). Climate change and temperature-dependent sex determination in reptiles. Proc. Natl. Acad. Sci..

[48] Shine R (2004). seasonal shifts in nest temperature can modify the phenotypes of hatchling lizards, regardless of overall mean incubation temperature. Funct. Ecol..

