# Supplementary material for: Effects of natural nest temperatures on sex reversal and sex ratios in an Australian alpine skink
Source: Sci Rep. 2021 Oct 11;11:20093. doi: 10.1038/s41598-021-99702-1 (PMC8505511; doi:10.1038/s41598-021-99702-1)
Supplement: Supplementary file 1 — Supplementary Information. [file 41598_2021_99702_MOESM1_ESM.pdf]

SCIENTIFIC REPORTS

SUPPLEMENTARY INFORMATION

Effects of natural nest temperatures on sex reversal and sex ratios in an Australian alpine skink

Duminda S. B. Dissanayake[1,2], Clare E. Holleley,[1,2] and Arthur Georges[1]
[1]Institute for Applied Ecology, University of Canberra, ACT 2601, Australia.
[2]Australian National Wildlife Collection, CSIRO, Canberra, ACT 2911, Australia.

Supplementary figures and tables

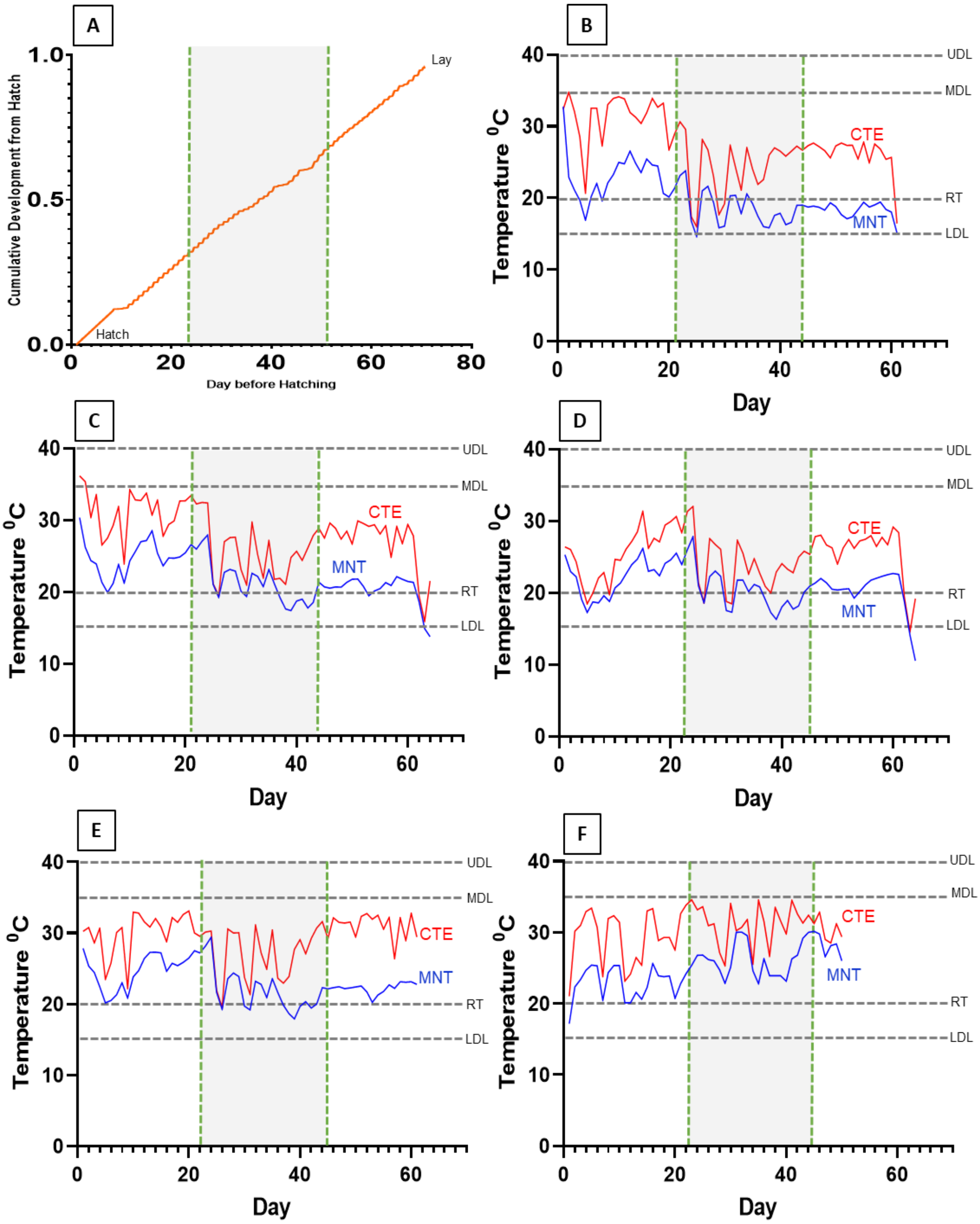

Figure S1. **(A)** Development of *Bassiana duperreyi* embryos under a temperature regime at the natural incubation environment; **B** to **F**: Temperature trace for the core of a nest of *B. duperreyi* showing traces for the mean nest temperature (MNT) and the constant-temperature equivalent (CTE) for the Dallwitz-Higgins model[32,33]. The threshold for sex determination (20˚C; Shine et al.[15]) (RT) and the thermosensitive period lies between lower development limit (LDL) and upper limit of development (UDL). Note that the thermosensitive period does not correspond to the middle third of incubation, either in position or duration, owing to the nonstationary trend in temperatures with season. Shaded area showing 33% to 67% of the thermosensitive period of each nest; **B**. Mt Ginini nest (Nest 66) with XXmale produced nest; **C**. Piccadilly circus nest (Nest 52) XXmale not recorded nest; **D**. Piccadilly circus nest (Nest 47) XXmale recorded nest; **E**. Cooma (Nest 76) nest XXmale recorded; **F**. Dartmouth nest (Nest 86) XXmale not recorded.

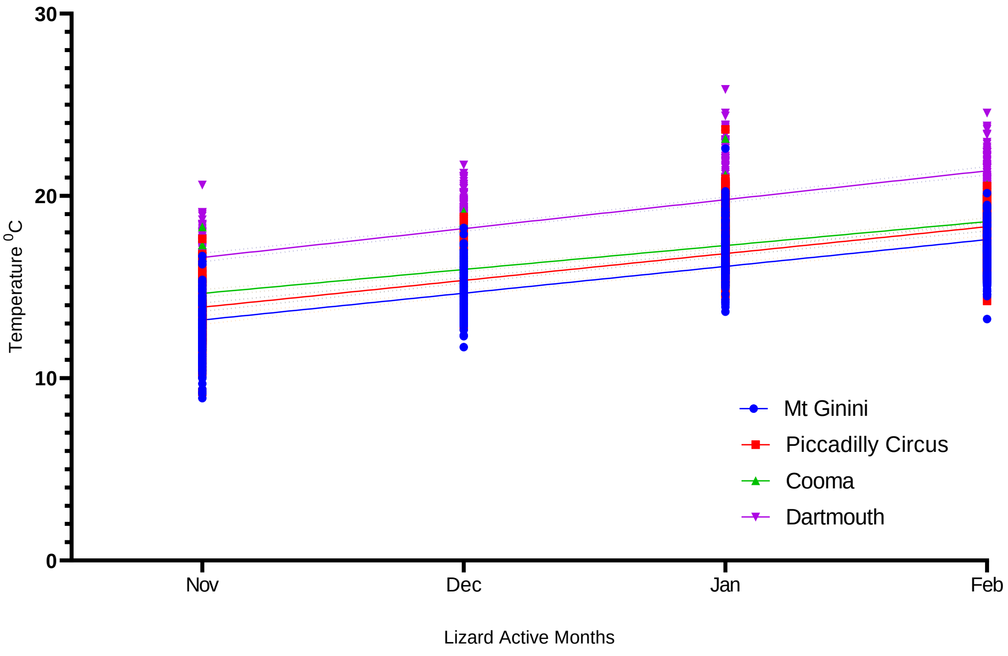

Figure S2. Mean air temperature during the skink active months (i.e., usually early November to late February, a 16-week period). Mean air temperature revealed a warming trend between 1889 to 2019[25].

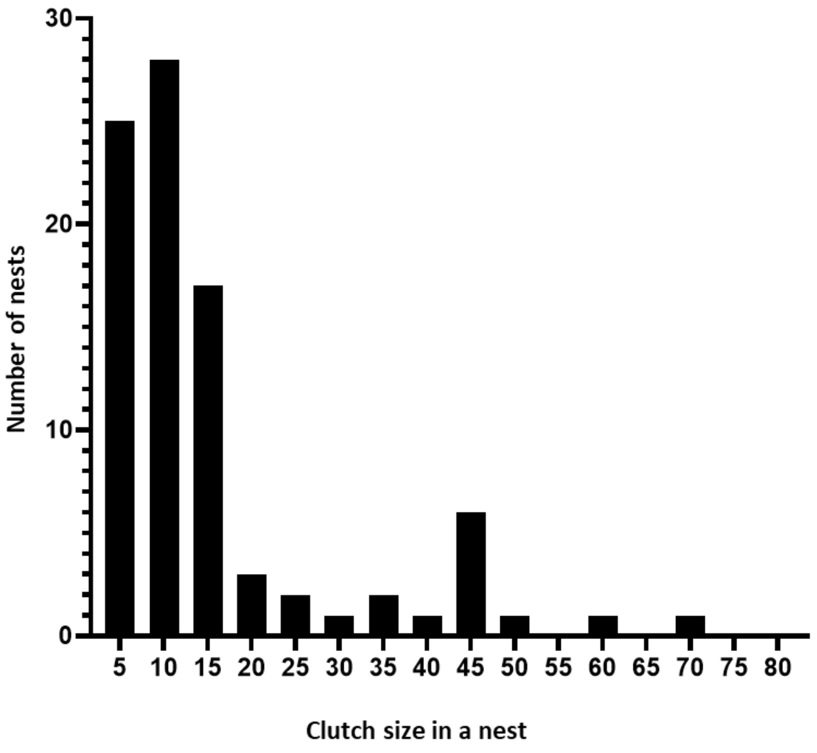

Figure S3. Number of eggs recorded in a nest during the study period.

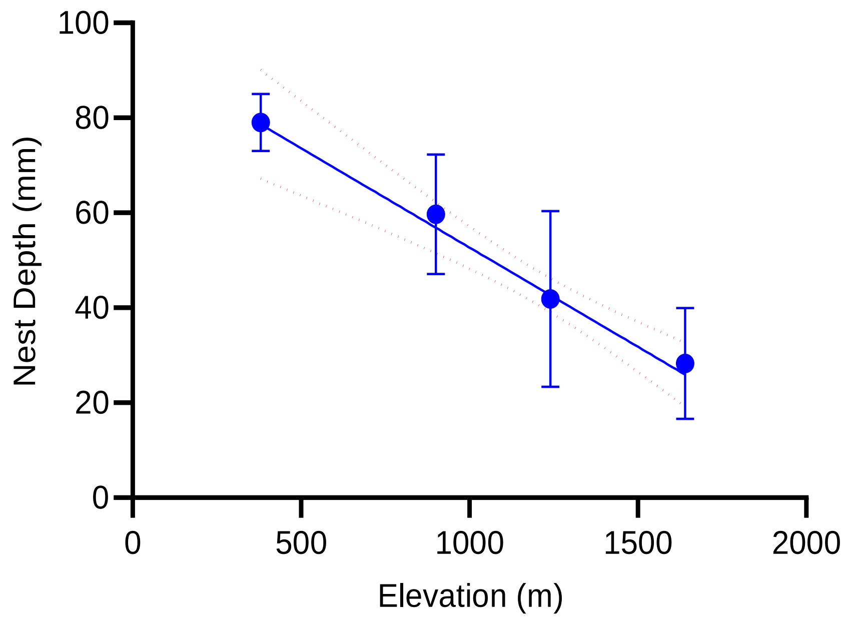

Figure S4: The pattern of increasing the mean nest depth with decreasing elevation.

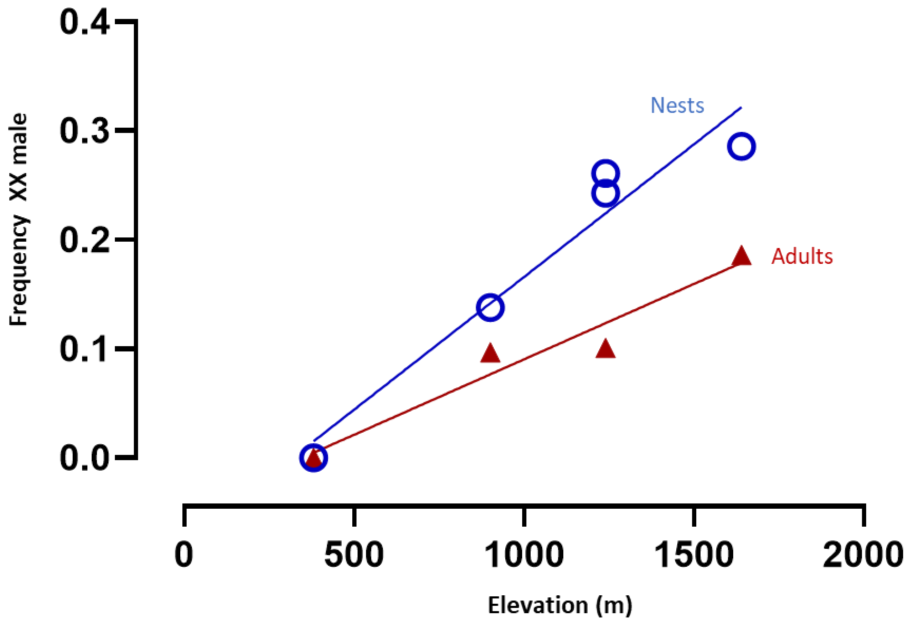

Figure S5: Frequency of XX males in two life stages in *B. duperreyi*. Adult frequency of sex reversal data from Dissanayake et al.[20]

Table S1. The mean weekly temperature regimes in two types of nets (sex reversed found nests and normal nests).

	Levene's Test for Equality of Variances		t-test for Equality of Means					
			t	df	Sig. (2-tailed)	Mean Difference ± SEM	95% Confidence Interval of the Differences	
	F	Sig.					Lower	Upper
Mt Ginini XXmale found nest vs XXmale not found nest	1.3	0.719	0.907	16	0.377	-0.9630 ± 1.061	-3.213	1.287
PCSI XXmale found nest vs XXmale not found nest	1.264	0.75	2.057	16	0.056	1.174 ± 0.5707	-0.03564	2.384
PCSII XXmale found nest vs XXmale not found nest	1.113	0.883	1.198	16	0.248	1.136 ± 0.9490	-0.8753	3.148
Cooma XXmale found nest vs XXmale not found nest	1.8	0.42	4.089	16	0.0009	1.922 ± 0.4701	0.9256	2.919

Table S2. Pearson r and P value for Pearson correlation

Correlation	Sex reversal frequency in the nests vs.							
	Elevation	Mean nest temperature	Tmax (°C)	Tmin (°C)	Total Rain (mm)	Evaporation (mm)	Radiation (MJ/m²)	Vapor pressure (hPa)
**Pearson r**	0.9797	-0.9574	-0.9952	-0.8223	0.3418	-0.9124	0.8474	-0.9874
95% confidence interval	0.3176 to 0.9996	-0.9991 to 0.04618	-0.9999 to -0.7848	-0.9961 to 0.6618	-0.9222 to 0.9807	-0.9982 to 0.3956	-0.6127 to 0.9967	-0.9997 to -0.5147
R squared	0.9597	0.9166	0.9905	0.6762	0.1168	0.8324	0.718	0.9749
**P value**								
P (two-tailed)	0.0203	0.0426	0.0048	0.1777	0.6582	0.0876	0.1526	0.0126
P value summary	*	*	**	ns	ns	ns	ns	*
Significant? (alpha = 0.05)	Yes	Yes	Yes	No	No	No	No	Yes
